# Translational Clinical Strategies for the Prevention of Gastrointestinal Tract Graft *Versus* Host Disease

**DOI:** 10.3389/fimmu.2021.779076

**Published:** 2021-11-26

**Authors:** Aditya Rayasam, William R. Drobyski

**Affiliations:** ^1^ Department of Medicine, Medical College of Wisconsin, Milwaukee, WI, United States; ^2^ Bone Marrow Transplant Program, Medical College of Wisconsin, Milwaukee, WI, United States

**Keywords:** graft versus host disease, inflammatory cytokines, gastrointestinal tract, translational clinical trials, allogeneic hematopoietic stem cell transplantation, mouse models

## Abstract

Graft versus host disease (GVHD) is the major non-relapse complication associated with allogeneic hematopoietic stem cell transplantation (HSCT). Unfortunately, GVHD occurs in roughly half of patients following this therapy and can induce severe life-threatening side effects and premature mortality. The pathophysiology of GVHD is driven by alloreactive donor T cells that induce a proinflammatory environment to cause pathological damage in the skin, gastrointestinal (GI) tract, lung, and liver during the acute phase of this disease. Recent work has demonstrated that the GI tract is a pivotal target organ and a primary driver of morbidity and mortality in patients. Prevention of this complication has therefore emerged as an important goal of prophylaxis strategies given the primacy of this tissue site in GVHD pathophysiology. In this review, we summarize foundational pre-clinical studies that have been conducted in animal models to prevent GI tract GVHD and examine the efficacy of these approaches upon subsequent translation into the clinic. Specifically, we focus on therapies designed to block inflammatory cytokine pathways, inhibit cellular trafficking of alloreactive donor T cells to the GI tract, and reconstitute impaired regulatory networks for the prevention of GVHD in the GI tract.

## Introduction

Graft versus host disease (GVHD) is the major non-relapse cause of morbidity and mortality occurring after allogenic hematopoietic stem cell transplantation (HSCT) (1, 2). GVHD consists of both acute and chronic phases, which have distinguishing temporal and pathophysiological characteristics (3–5). Acute GVHD primarily targets the skin, gastrointestinal (GI) tract, lung, and liver, with the GI tract being the primary target organ that determines subsequent morbidity in patients (6). Involvement of this tissue site can be attributed to the conditioning regimen that licenses the gut to release damage (DAMPS) and pattern-associated molecular patterns (PAMPs) that activate and recruit innate immune cells (7). These cells then lead to the activation and clonal expansion of alloreactive T cells, which perpetuate a proinflammatory cascade that ultimately results in pathological damage (8). Ultimately, GVHD in the GI tract can result in protracted immune suppression, infectious complications due to compromised mucosal integrity, and prolonged hospitalization.

Corticosteroids have long been first line therapy for patients with acute GVHD in the GI tract as they function to inhibit inflammatory pathways and cytokine production (9, 10). However, clinical responses do not occur in all patients as up to 50% can become refractory to systemic therapy (11). In addition, corticosteroids have side effects, which can be disabling and life threatening, including diabetes, infectious complications, and myopathy (12). For these patients, secondary agents for steroid resistant disease are much less effective and mortality is unacceptably high. Thus, prevention of this complication has emerged as a primary goal in the field in order to circumvent the need for prolonged immune suppressive therapy in patients who develop GVHD in this tissue site.

**Figure 1:**
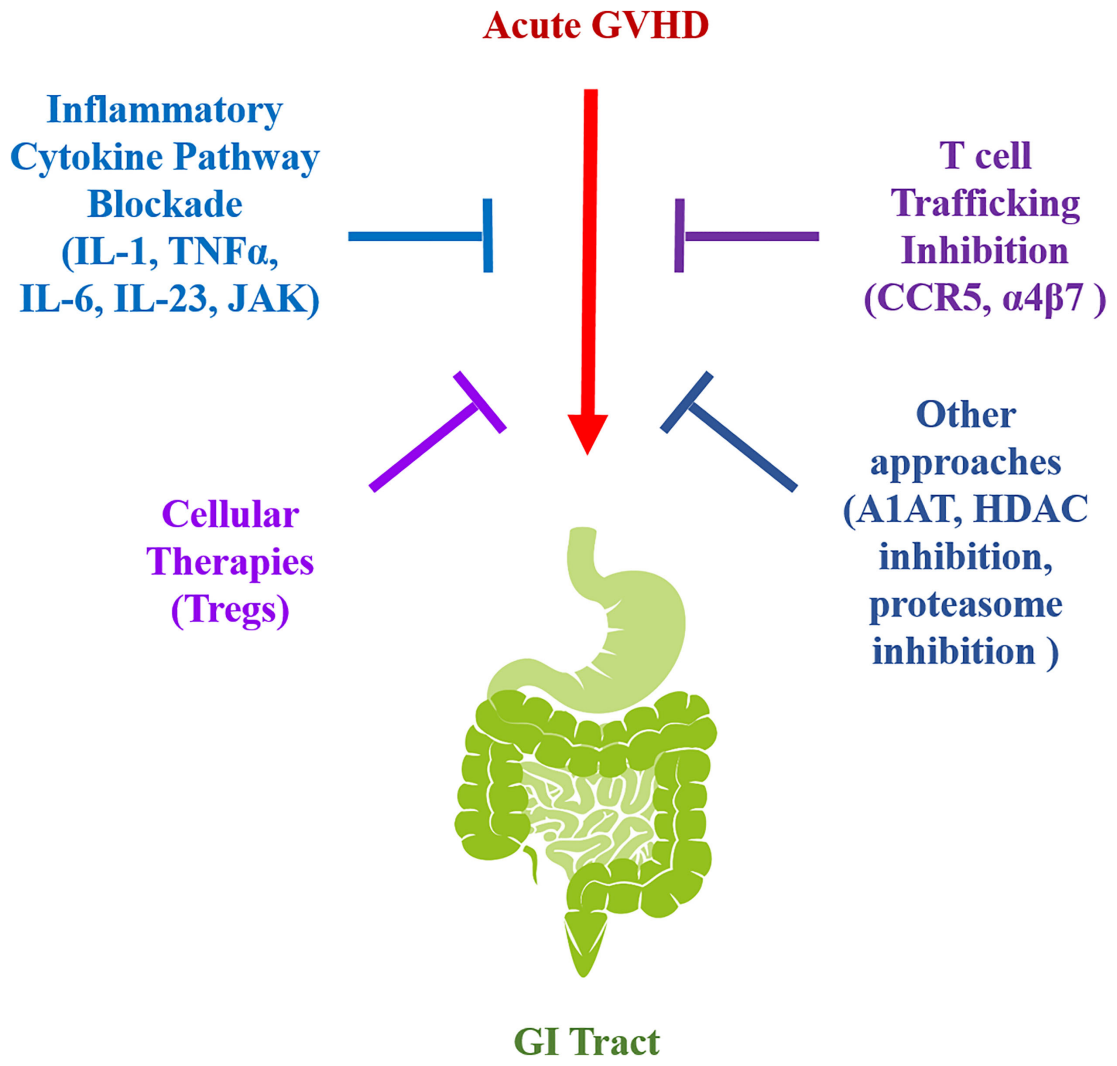
Graphical schematic summarizing the translational strategies for GI GVHD prevention.

Amelioration of this complication in humans is therefore dependent upon increasing our understanding of the pathophysiology of GI GVHD. To unravel pathophysiologic mechanisms by which this disease is propagated and devise potentially translatable clinical strategies, animal models, primarily using mice, have been employed to examine how dysregulation of the immune system occurs in this setting (7, 13–16). From this work, a number of strategies have been examined that include the blockade of inflammatory cytokine pathways, the alteration of T cell trafficking into the GI tract, and the re-establishment of competent regulatory mechanisms (**Figure 1**). In this review, we highlight recent pre-clinical studies in each of these areas and examine the results from the subsequent clinical trials that have emerged as a direct translation of this work in human allogeneic HSCT recipients.

## Blockade of Inflammatory Cytokine Pathways

### Interleukin 1

Interleukin-1 (IL-1) was the first interleukin to be described and exhibits a myriad of functions that are critical for inflammation. IL-1, along with 10 other members, comprise the IL-1 super family since they possess a highly conserved gene structure and are primarily clustered in a 400kb region of human chromosome 2 (17, 18). While primarily relevant for promoting the activity of innate immune myeloid cells, IL-1 also plays a key role in the differentiation of T_H_17 cells (19). The role of IL-1 has been explored preclinically in several immune-mediated diseases such as inflammatory bowel disease, asthma, and psoriasis but has mainly been tested clinically in rheumatoid arthritis with modest results (20).

McCarthy et al. first demonstrated that IL-1 could be a critical modulator of acute GVHD in murine studies. IL-1α was observed to be elevated in the skin of mice with GVHD and inhibition of IL-1 signaling with an IL-1R antagonist reduced GVHD mortality without impairing engraftment (21). Subsequently, Abhyankar et al. revealed that mRNA levels of IL-1 transcripts were increased several hundred-fold in GVHD target organs and also reported that IL-1R antagonist treatment could reduce mortality (22). Unfortunately, a later study showed only transient benefits of IL-1R antagonists in a minor antigen-disparate murine model and no effects in a fully MHC disparate model (23), suggesting that other pro-inflammatory cytokines may be able to compensate for deficiencies in IL-1 signaling during acute GVHD.

More recently, Park and colleagues evaluated the mechanism for how IL-1 blockade alleviates GVHD severity (24). They demonstrated that pretreatment of donor cells with an IL-1R antibody increased the proportion of Tregs to Th17 cells in host organs. Moreover, they observed decreased numbers of T cells and improved pathology in the GI tract, implicating a role for IL-1 in mediating intestinal inflammation during GVHD. In addition, Jankovic et al. demonstrated that early blockade of IL-1β as well as genetic deficiency of IL-1R in donor dendritic cells and T cells both improved GVHD-induced mortality (25). Correspondingly, immunohistochemical staining with IL-1β of intestinal biopsies revealed that the density of IL-1β staining correlated with augmented GVHD grades in patients. Altogether, these preclinical data suggested that IL-1 antagonism could improve GI GVHD by influencing donor T cell phenotypes and infiltration into the gut.

Based on preclinical data indicating that IL-1 inhibition could be beneficial for GVHD, Antin et al. tested whether treatment with a recombinant human IL-1 receptor antagonist could be beneficial for the prevention of acute GVHD in a phase I/II trial. They observed stage-specific improvements of GVHD in the skin, liver and particularly the GI tract (82% of patients) as well as demonstrated that the treatment was safe (26). Another phase I/II clinical study also showed improvements in 8/14 GVHD patients treated with a recombinant human IL-1 receptor antagonist, but only 33% of patients with GI-tract involvement displayed improvement (27). Due to these preliminary results, Antin et al. performed a larger scale double-blind, randomized, placebo-controlled study including 186 patients who underwent allogenic stem cell transplantation. Either IL-1R antagonist or placebo was given to patients from day -4 to 10 days after conditioning with cyclophosphamide and total body irradiation. Disappointingly, they found that there was no statistically significant difference in the percentage of the patients in the IL-1R antagonist versus placebo group that developed moderate to severe GVHD. Furthermore, there was no difference in hematologic recovery, toxicity, or overall survival (28). Based largely on these negative results, specific inhibition of IL-1 signaling has largely been abandoned as a therapeutic approach to prevent GVHD globally and more specifically in the GI tract.

### Tumor Necrosis Factor-Alpha

TNF-α, a pleiotropic inflammatory cytokine involved in the pathogenesis of rheumatoid arthritis, psoriasis, inflammatory bowel disease, and cancer also plays a role in acute GVHD. While TNFα is primarily produced by macrophages and monocytes during acute inflammation, context-dependent insults can induce TNFα to be released by lymphocytes, endothelial cells and other cell types as well. TNFα signaling occurs through 2 receptors; TNFR1, which is ubiquitously expressed and promotes inflammation and tissue damage, and TNFR2, which is restricted to a few cell types and responsible for homeostatic functions (29).

One of the first pre-clinical studies to evaluate the role of TNFα in GVHD was performed in 1987. Piguet et al. utilized a semi-allogenic murine model to assess whether administration of anti-TNFα antibodies eight days after GVHD induction could be beneficial. This therapeutic approach limited skin epidermal necrosis, reduced gut epithelial cell damage, and decreased mortality (30). The authors attributed the inflammatory effects of TNFα in the GI tract to be mediated by donor lymphocytes and potentially due to the increase of Ia expression on the gut mucosal epithelium. Moreover, pathological analysis revealed that TNFα induced gut dilatation with marked flattening of the villi and elevation of the crypts (30). More recent studies have expanded upon those initial results to help define the mechanisms of TNF-α during different stages of GVHD. Schmaltz et al. demonstrated that allogeneic T cells deficient in TNF induced significantly less morbidity and mortality compared to control T cells. Moreover, TNF deficiency in donor T cells induced reduced histological damage in the lower GI tract (31). Additional mechanistic studies by Stickel et al. demonstrated that miR-146a regulates the transcription of TNF levels and that T cells deficient in miR-146a induced augmented levels of TNF-α and worsened GVHD severity. Correspondingly, overexpression of miR-146a in donor T cells reduced TNFα levels and pathological damage in the small intestine and colon (32). Collectively, these preclinical studies provided rationale for utilizing anti-TNF therapies to treat GVHD patients with GI tract involvement.

Early clinical studies helped to define the kinetics of TNFα production following GVHD. This work demonstrated that systemic TNFα levels were increased during the conditioning phase (33) as well as early post transplantation (34, 35). Moreover, Holler et al. demonstrated that augmented levels of TNFα preceded complications of bone marrow transplantation and correlated with the development of acute GVHD symptoms, indicating that anti-TNFα therapy could be a promising option for GVHD prophylaxis.

Administration of infliximab, a chimeric IgG1 monoclonal antibody which binds to soluble and transmembrane human TNF-α, has been given to patients for GVHD treatment with some success (36), but there is limited clinical data on the efficacy of infliximab for GVHD prophylaxis, particularly in the GI tract. Hamadani et al. conducted a prospective trial of infliximab for the prophylaxis of GVHD (37). Infliximab or placebo was administered one day prior to conditioning, but unfortunately treated patients exhibited similar incidences of grade II-IV acute GVHD compared to the control group (both ~36%). Choi et al. performed a phase II clinical trial at two centers to test whether etanercept, which is a soluble receptor that binds to both TNFα and TNFβ, could reduce TNFR1 levels, ameliorate GVHD occurrence and improve survival (38). Surprisingly, etanercept did not influence TNFR1 levels in patients who received TBI-based conditioning but was rather effective in patients who received non-TBI based regiments. Etanercept treated patients who were not conditioned with TBI exhibited relatively low rates of grade III or IV GVHD (16%). Moreover, they reported that lower TNFR1 levels correlated with GVHD mortality. Unfortunately, this study did not test whether etanercept ameliorated the severity of GI GVHD. Overall, there have been only limited and inconclusive data that TNFα targeting strategies are efficacious for acute GVHD and none evaluating the prevention of GI GVHD.

### Interleukin 6

IL-6 is a pleiotropic cytokine and plays a critical role in regulating acute and chronic inflammation, hematopoiesis, metabolic control, and metabolism. IL-6 can be produced by a variety of cells including fibroblasts, muscle cells, keratinocytes, monocytes, macrophages, and endothelial cells (39). During acute inflammation, monocytes and macrophages rapidly produce IL-6 in response to PAMPs and DAMPs. Moreover, IL-6 contributes to the differentiation of T_H_17 cells and plays an integral role in skewing naïve T cells towards proinflammatory phenotypes limiting regulatory T cell (Treg) differentiation. Several studies have identified a role for IL-6 and members of the IL-6 superfamily (IL-11, IL-23, IL-27, and IL-31) in contributing to autoimmune disorders, cancer, and GVHD (40–42).

Pre-clinical murine studies by Chen et al. demonstrated that antibody-mediated blockade of IL-6R reduced pathologic damage associated with GVHD. Specifically, histological analyses revealed that IL-6R inhibition had a profound effect on minimizing inflammation within the colon. Mechanistically, inhibition of IL-6 signaling augmented the generation of Tregs and correspondingly reduced T_H_1 and T_H_17 cell expansion (42). Interestingly, the colon displayed the highest levels of IL-6 and IL-6R expression after GVHD. Both donor and host production of IL-6 appeared to be important as transplantation with IL-6^−/−^ recipient or donor mice had no protective effect on GVHD mortality. While Chen et al. demonstrated the importance of IL-6 in both the donor and recipient directions, another report observed that IL-6 deficiency in donor T cells was sufficient to protect mice from the effects of GVHD (43). The experimental designs of these studies however differed with respect to radiation dose, length of IL-6 inhibition, and purity of T cells in the transplant inoculum. This study also confirmed that administration of an anti-IL-6R antibody protected animals from lethal GVHD and reduced pathological damage in the GI tract, although there was no effect on Treg reconstitution.

Recently, the role of IL-6 during the pathophysiology of acute GVHD was further defined (44). This study sought to identify the cell types responsible for IL-6 signaling that perpetuate gut-associated GVHD. The authors conducted studies in which the IL-6R was specifically deleted from intestinal cells using Villin-Cre mice. They observed that this had no effect on acute GVHD pathology in the GI tract indicating that IL-6R expression in the GI tract was dispensable. Rather, subsequent experiments revealed that IL-6 secretion by recipient DCs was critical for initiating GVHD by way of classical signaling upon interactions with donor T cells. In fact, deletion of DC produced IL-6 specifically prevented the differentiation of pro-inflammatory donor T_H_17 and T_H_22 cells and subsequent damage to the GI tract. Overall, this study further confirmed a role for IL-6 in acute GVHD pathophysiology in the GI tract.

Tocilizumab is a humanized monoclonal antibody that binds to both membrane-bound and soluble forms of the IL-6R and was initially approved for the therapy of patients with rheumatoid arthritis (45). Based on preclinical data, studies sought to determine whether the prophylactic administration of tocilizumab could prevent the development of lower GI tract GVHD. To that end, a study from Australia showed that administration of tocilizumab in addition to standard immune suppression resulted in a very low incidence of both grades II-IV (12%) and III-IV (4%) acute GVHD. There was also a low incidence of GI tract involvement (8%) reported in a heterogeneous group of patients that included those that received reduced intensity and myeloablative conditioning regimens (46). A subsequent phase II trial designed in similar fashion to that of Kennedy and colleagues also administered a single dose of tocilizumab as prophylaxis to patients that also received standard immune suppression. Following treatment with tocilizumab, only 3% and 6% of patients displayed grade III-IV acute GVHD by days 100 and 180, respectively. Importantly, no patient developed lower GI tract disease within the first 100 days, providing evidence that tocilizumab was effective for the prevention of GI tract GVHD in humans (47).

A more recent phase III trial administered standard immune suppression plus either tocilizumab versus placebo to a heterogeneous group of patients in Australia. Patients received either reduced intensity or myeloablative conditioning regimens followed by transplantation of peripheral stem cell grafts from matched sibling or unrelated donors. The results of this study showed a non-significant trend towards improvements in grade II-IV acute GVHD and acute GVHD-free survival. There were no statistically significant reductions in moderate to severe GVHD in any specific tissue sites, including the GI tract, although there was a trend towards more favorable outcomes in tocilizumab-treated patients. Limitations of the study were the lack of a centralized GVHD grading committee across all centers, the fact that the control group fared much better than in earlier publications with respect to acute GVHD-free survival, and concerns that the study was under powered to detect more modest differences in experimental end points (48). Collectively, these studies support further research designed to determine whether blockade of IL-6 signaling is efficacious for the prevention of GVHD within the GI tract in humans.

### Interleukin 23

IL-23 is a pro-inflammatory cytokine that is a member of the IL-12 family that includes IL-27, IL-35, and IL-39 and is primarily produced by dendritic cells and macrophage/monocyte populations. IL-23 regulates T cell and natural killer cell responses as well as induces the differentiation of T_H_1 cells and prolongs their survival. IL-23 shares a p40 subunit with IL-12 but also has a unique p19 subunit as well. Members of the IL-12 family have been demonstrated to play a pro-inflammatory role in autoimmunity as well as bacterial and parasite-induced infections (49).

With respect to GVHD pathophysiology, several reports have identified that inhibition of IL-23 signaling with either antibody-based or genetic strategies reduces the severity of GVHD without compromising GVL effects in murine transplantation models. Importantly, these studies demonstrated that there was preferential protection from pathological damage within the GI tract (50, 51). These findings indicated that IL-23 has an important organ-specific role within the context of a systemic inflammatory disorder. More recently, additional studies demonstrated that blockade of the IL-23 receptor (IL-23R) by either antibody or genetic approaches also reduced overall GVHD mortality and protected animals from pathological damage in the GI tract (52). This was attributable to a population of CD4^+^ IL-23R^+^ T cells that directly mediated tissue damage. Further examination uncovered a subset of CD4^+^ T cells that not only co-expressed the IL-23R but also express the beta 2 integrin CD11c and gut homing molecules α4β7 and CCR9. These cells constituted a colitogenic CD4^+^ T cell population that possessed an innate-like gene signature, suggesting that these cells serve as an important bridge between the innate and adaptive arms of the immune system and are positioned to mediate early inflammatory events. More recently, Bastian et al. confirmed that IL-23R alpha was required for the induction of GVHD development and that absence of IL-23R signaling in both CD4^+^ and CD8^+^ T cells resulted in a decrease in the production of GM-CSF and IFN-γ in the GI tract, further corroborating the importance of IL-23 signaling during acute GVHD (53).

From a translational perspective, ustekinumab which blocks the common p40 subunit shared by IL-12 and IL-23 has been administered to allogeneic HSCT recipients to prevent GVHD. In a randomized, blinded, placebo-controlled trial, Pidala and colleagues demonstrated that ustekinumab was effective in suppressing IL-12/IL23p40 levels and reducing the levels of IL-17 and IFN-alpha. However, ustekunimab-treated patients had no difference in the incidence of grades II-IV acute or chronic GVHD, and there was no specific protective effect noted in the GI tract (54). Another follow-up randomized trial to address this question and determine whether this antibody can prevent GVHD has recently opened (NCT04572815). While there have not been any published studies that examined whether selective blockade of IL-23 can mitigate the severity of GVHD, a phase I-II clinical trial utilizing the p19-specific antibody tildrakizumab is currently under way (NCT04112810).

### Janus Kinase Inhibition

The JAK-STAT pathway involves a family of intracellular tyrosine kinases that regulate the function of key inflammatory cytokine signaling pathways (55). This family includes four JAK and seven STAT proteins which together respond to cues outside of the nucleus to ultimately facilitate transcription of immune-related genes responsible for regulating inflammation (Aaronson et al., 2002 Science).

Several preclinical studies have demonstrated a role for JAK-STAT signaling in mediating cytokine release and inducing GVHD target organ damage (56–58). For example, Ma et al. showed that abrogating JAK/STAT1 signaling in donor T cells could ameliorate GVHD and that transplantation of Stat1-deficient donor cells resulted in enhanced protection in the small intestine and colon (57). Subsequent studies substantiated that work by demonstrating that pharmacological inhibition of JAK1/2 with ruxolitinib could reduce GVHD while preserving graft versus tumor responses (59, 60). Carniti and colleagues observed that ruxolitnib improved overall survival and reduced pathological damage in target organs that included the small and large intestine. Protection in the GI tract was attributable to a reduction in T cell and macrophage infiltration that was due, in part, to reduced CXCR3 expression on allogeneic T cells (60).

Other JAK inhibitors have also been utilized in pre-clinical studies to prevent acute GVHD. Choi et al. administered baricitinib, a selective inhibitor of JAK1 and JAK2, and demonstrated that this agent could prevent GVHD by expanding the Treg pool and downregulating CXCR3 expression on T_H_1 and T_H_2 cells (61). Interestingly, baricitinib was superior to ruxolitnib in preventing GVHD-induced mortality. More recently, Sun and colleagues utilized a highly selective JAK1 inhibitor (SHR0302) (62) and demonstrated improved overall survival when compared to vehicle treated controls (63). SHR0302 also reduced the infiltration of immune cells into the GI tract through reduction of CXCR3 expression on donor T cells as well as mitigated the release of the proinflammatory cytokines, IL-6, IFN-γ, and TNF-α.

Cumulative preclinical work and the success of JAK inhibitors as salvage therapy for GVHD treatment in patients (64, 65) ultimately led to the FDA approval of ruxolitnib for the treatment of steroid refractory acute GVHD (66). This success has also been the impetus for clinical trials designed to assess whether JAK inhibition could be successful for GVHD prophylaxis. To that end, a recent trial revealed that the JAK1 inhibitor itacitinib was well tolerated and displayed efficacy in steroid refractory acute GVHD (67). Consequently, there are now several ongoing trials designed to examine the efficacy of itacitinib for acute GVHD prophylaxis (NCT04339101), (NCT03755414) and (NCT04859946). Results from these trials should help delineate whether JAK targeting strategies are efficacious for acute GVHD prophylaxis and if administration of this class of agents prevents pathological damage in the GI tract.

## Inhibition of T Cell Trafficking

### CCR5

Trafficking of donor T cells into the GI tract and the establishment of tissue residency have been shown to be critical events in the pathophysiology of GVHD in this tissue site (68). Consequently, strategies to prevent donor T cell trafficking into the GI tract have been examined as an approach to mitigate pathological damage. CCR5 is a chemokine primarily expressed on the surfaces of T cells, NK cells, and macrophages. It facilitates immune cell trafficking through the cognate ligands CCL3, CCL4 and CCL5, which can be expressed in inflammatory sites. Several studies have identified that CCR5 facilitates migration of memory CD8 T cells during viral infections (69, 70), Tregs in tumor progression (71), and NK cells in murine models of hepatitis (72).

In transplantation studies, Murai et al. demonstrated in a parent to F1 model that disrupting a gene encoding CCR5 could prevent the recruitment of donor T cells into Peyer’s patches (PPs) and reduce acute GVHD. They concluded that donor cytotoxic T cells utilize CCR5 to enter the gut and that the PP is an essential site for initiating GVHD (73). Conversely, Welniak and colleagues showed that transplantation of CCR5 knockout donor cells into lethally irradiated MHC-mismatched recipients increased T cell produced IFNγ and TNFα in the GI tract and induced pathological damage in the gut (74). In a subsequent study, Wysocki et al. identified a critical role for CCR5 expression on donor CD4^+^ CD25^+^ Tregs. Specifically, CCR5 expression on donor Tregs seemed to be essential for entry into the lung, liver, spleen, and mesenteric lymph nodes (75). Collectively, these results suggest that the role of CCR5 during GVHD appear to be model and perhaps cell dependent.

Reshef et al. examined the effect of the CCR5 antagonist, maraviroc, on lymphocyte function and chemotaxis *in vitro* as well as performed a phase 1/2 study on 38 high-risk patients who received standard immune suppression along with maraviroc as GVHD prophylaxis (76). They observed that maraviroc inhibited lymphocyte chemotaxis and noted a low incidence of grades II to IV acute GVHD (15 and 24% on days 100 and 180, respectively). Only 9% of patients developed GVHD in the GI tract within the first year. Moy et al. also demonstrated that maraviroc treatment resulted in a lower incidence of acute GVHD and reduced levels of the gut-specific marker Reg3a, which is associated with epithelial integrity (77). More recently, Reshef et al. performed a subsequent phase II trial to examine the efficacy of an extended course of maraviroc in 37 patients. They found that the rate of grade II-IV acute GVHD was 22 ± 7% and the grade III-IV acute GVHD was 5 ± 4% at 180 days, while noting that GVHD of the GI tract was uncommon (78). They concluded that compared to the prior short-course treatment study, an extended course of maraviroc could result in significantly higher GVHD-free, relapse-free survival. The requirement for an extended course of maraviroc suggested that more prolonged inhibition of CCR5 signaling might be required for durable prevention of GVHD of the GI tract. Despite those promising clinical results, however, a recent trial evaluating maraviroc for GVHD prophylaxis did not demonstrate superior protection from acute GVHD when combined with standard immune suppression. Specifically, Bolaños-Meade et al. conducted a randomized phase II trial in which one of the arms examined the efficacy of maraviroc, tacrolimus and methotrexate as GVHD prophylaxis (79). This studied revealed that there was no difference in the incidence of grade III or IV acute GVHD or overall survival in these patients when compared to those treated with tacrolimus and methotrexate alone, which represented the control group. Whether a more extended course of maraviroc could be required to achieve GVHD prophylaxis in the GI tract in some patients has not been formally examined in a randomized setting.

### α4β7 (Lymphocyte Peyer Patch Adhesion Molecule)

Lymphocyte Peyer patch adhesion molecule (LPAM), also known as α4β7 integrin, is responsible for homing into gut-associated lymphoid tissue. When expressed on T lymphocytes, α4β7 integrin licenses cells to bind to mucosal addressin cell adhesion molecule (MAdCAM), which is chiefly expressed on high endothelial venules of mucosal lymphoid organs as well as intestinal lamina propria (80). Given the importance of this ligand/receptor interaction, numerous investigators have explored the role of α4β7 integrin in propagating GVHD, particularly with regards to inflammation in the GI tract.

Several pre-clinical studies have been conducted to examine the role of α4β7/MAdCAM in the pathophysiology of GVHD. Petrovic et al. showed that transplantation of allogeneic α4β7^−/−^ T cells resulted in significantly reduced GVHD-induced mortality compared to wild type T cells which was attributed to delayed homing to the intestines and liver (81). In addition, Waldman et al. also explored the role of α4β7 in GVHD by transplanting β7-deficient allogeneic T cells into conditioned mice. Despite β7-deficient T cells having intact activation, proliferation, cytokine production, and cytotoxicity, they induced less GVHD morbidity and mortality compared to wild type T cells due to their inability to traffic to the liver and the gut (82). Utilizing an MHC-mismatched murine transplantation model, Dutt and colleagues demonstrated that genetic deletion of α4β7 integrin alone was insufficient to protect mice from lethal GVHD; but rather the deletion of both α4β7 and CD62L together were required to protect mice from GVHD (83). This study suggests that α4β7 and L-selectin may have additive effects in influencing T cell homing to the gut. Another report demonstrated that inhibition of MAdCAM-1 reduced the recruitment of donor CD8^+^ T cells into the intestine and alleviated GVHD by limiting intestinal injury (84). They also demonstrated that delayed administration of an anti-MAdCAM-1 antibody reduced intestine-infiltrating α4β7^+^ CD8^+^ T cells without compromising anti-leukemic effects. Collectively, these studies indicated that both CD4 and CD8 cells utilize α4β7 integrin to enter the GI tract during GVHD. Recent work by Fu and colleagues utilized 3D imaging to visualize intricate allogeneic T cell spatial localization within the GI tract following GVHD (85). These data demonstrated that intestinal stem cells were the primary target of alloreactive donor T cells. Moreover, they demonstrated that this process is dependent on β7 integrin and MAdCAM-1 interactions as inhibition with anti-MAdCAM-1 antibody reduced donor T cell invasion into the lower crypt regions of the mucosa and attenuated GI tract damage.

In clinical studies, Chen et al. examined the peripheral blood of patients with symptoms of acute GVHD before treatment (86). The collected samples were subcategorized into three groups: intestinal GVHD, skin GVHD, and no GVHD. Interestingly, they reported that patients with intestinal GVHD had a significantly higher percentage of α4β7 integrin-expressing memory CD8^+^ T cells (7.7%) compared to patients with skin GVHD (1.3%) and no GVHD (1.0%). α4β7 was not differentially expressed on any CD4^+^ or CD8^+^ T cell subsets that were analyzed. Therefore, this study highlights the importance of α4β7 expression on CD8^+^ T cells particularly for propagating human GVHD symptoms within the gut.

Vedolizumab, a monoclonal antibody that binds to α4β7, has been approved for treatment of ulcerative colitis and Crohn’s disease and, more recently, has been examined as a treatment for steroid refractory GI GVHD with variable results (87, 88). Danylesko et al. retrospectively analyzed the efficacy of vedolizumab in 29 patients from three transplant centers, 24 of which displayed histopathology associated with gut GVHD (89). An overall response rate of 79% was observed with 28% of patients having a complete response, despite treatment being administered mainly as second- or third-line therapy. Notably, a large percentage (69%) of patients who received early administration of vedolizumab were able to have immunosuppression discontinued altogether, supporting the premise that vedolizumab was most effective for patients with steroid refractory severe GI GVHD when administered soon after onset. Recent findings from Mehta et al. substantiated this conclusion that early treatment with vedolizumab for GVHD may be necessary for optimal results as vedolizumab treatment as a secondary or tertiary treatment for grade III of IV patients who were refractory to ruxolitinib, displayed minimal response rates (90). More recently, Fløisand et al. conducted another clinical trial to evaluate the efficacy of vedolizumab for steroid refractory intestinal GVHD and observed a response rate in over two-thirds of participants (91). Unfortunately, the study did not evaluate GVHD prophylaxis and had to be discontinued prematurely as vedolizumab did not meet the primary efficacy endpoint at 28 days.

These data demonstrate that delayed α4β7 inhibition has limited efficacy for the treatment of acute GVHD patients who are steroid refractory. This could be due to the fact that there are multiple trafficking mechanisms employed by allogeneic T cells that contribute to lower GI tract damage, or that pathological damage facilitated by T cell entry into the gut occurs rapidly after transplant and may be dispensable at later time points. Cumulatively, these data suggest that earlier intervention may be necessary and that vedolizumab may be better suited as a preventive therapy rather than as steroid-refractory secondary treatment for gut-associated acute GVHD. To that end, there is currently a trial evaluating the efficacy of vedolizumab for acute GVHD prophylaxis (NCT03657160). This trial will assess the effect of vedolizumab on decreasing the incidence of GI-acute GVHD and acute GVHD-induced mortality 6 months after transplant. Results from this study will help to determine whether administration of vedolizumab could be appropriate for GI GVHD prevention.

## Cellular Therapy

### Regulatory T Cells

Regulatory CD4^+^ T cells are immunosuppressive lymphocytes that express high levels of the IL-2 receptor alpha-chain CD25, as well as the fork-head box transcription factor, Foxp3 (92). Natural Tregs (nTregs) arise in the thymus and comprise a small percentage of the total CD4^+^ T cell population that is present in the periphery. These cells are responsible for maintaining immune homeostasis and promoting tolerance to self-antigens to prevent autoimmunity (93). Due to their low frequency, it can be difficult to obtain high numbers of nTregs. However, Tregs can also be induced (iTregs) in the presence of TGF-β and IL-2 from conventional CD4^+^ T cells and have been employed to mitigate inflammation caused by effector T cells (94, 95). While it remains a challenge to maintain the immunosuppressive functions of iTregs *in vivo*, the relative ease of expansion and potent anti-inflammatory properties have generated interest in elucidating their potential therapeutic role for GVHD (96).

Taylor et al. performed experiments to deplete CD4^+^ CD25^+^ before allogeneic T cell transfer and to deplete Tregs *in vivo* by administering a CD25-depleting antibody (97). Both depletion strategies increased allogeneic T cell mediated GVHD. Moreover, transplant of cultured CD4^+^ CD25^+^ cells with allogeneic T cells before transplant significantly inhibited lethal GVHD *in vivo*. Subsequently, they demonstrated that high levels of L-selectin on Tregs were required for them to inhibit allogeneic T cell responses and limit GVHD (98). Importantly, Tregs that prevented GVHD did not interfere with GVL effects (99). Tawara et al. identified that IL-10, the major anti-inflammatory cytokine produced by Tregs, did not prevent disease or pathology in the gut when administered exogenously, but that Treg-derived IL-10 was able to induce GI protection and improve mortality (100). Moreover, they demonstrated that host APCs are required to facilitate the expansion of donor regulatory IL-10 producing T cells during GVHD and yield benefits in the GI tract (101).

Brunstein et al. conducted a study in which they enriched CD4^+^ CD25^+^ Foxp3^+^ cells from umbilical cord blood before transplantation into 23 acute GVHD patients (102). They observed that patients treated with these cells had reduced levels of grade II-IV GVHD patients compared to those that did not receive Treg therapy (43% versus 61%). The same group performed a similar study that resulted in only 9% of treated patients developing grade II-IV acute GVHD at 100 days compared to 45% in control patients. Contemporaneously, Di Ianni and colleagues evaluated whether infusion of donor CD4^+^ CD25^+^ Tregs could prevent acute GVHD in patients who received haploidentical transplants (103). Strikingly, of the 28 patients who received transplants, 26 achieved engraftment and only two developed grade III or IV GVHD. Unfortunately, neither of these studies analyzed organ-specific effects.

More recently, Meyer et al. performed a phase I/II study to test whether administration of human leukocyte-matched Tregs with CD34-selected hematopoietic cells and conventional T cells could prevent acute GVHD in patients undergoing myeloablative HCTs for hematological malignancies (104). They reported that of the 12 patients who received highly pure cryopreserved (n=5) or fresh (n=7) Tregs (<90%), only two acquired grade III or IV GVHD, with only one developing GI GVHD. Interestingly, none of the seven patients who received fresh Tregs developed acute or chronic GVHD, suggesting that fresh cells may be more efficacious for transplant. While findings from this trial are promising, the small number of patients in this study make it difficult to draw meaningful conclusions. Notably, the same group has a follow up trial underway that should involve more patients (NCT04013685).

Macmillan performed a phase I study to determine the safety and efficacy of induced Tregs (iTregs) on GVHD prophylaxis in adults with high-risk malignancy (105). They reported that iTregs could be safely infused into the adults and circulated for up to multiple weeks. Only three out of 14 patients developed acute GVHD with one experiencing grade IV lower GI involvement following transplant. Moreover, they found that 11% of the iTregs were CD103^+^, which is noteworthy given that CD103 is an integrin that is associated with gut homing in T cells. While these trials show some promise, optimizing the ability of Tregs to maintain their functions *in vivo* under inflammatory conditions and improving their gut-homing capabilities will be critical for preventing GI tract acute GVHD. Currently there is an active phase I trial to administer *ex-vivo* expanded donor regulatory cells for the prevention of acute GVHD (NCT01795573). This trial is designed to co-culture recipient dendritic cells and donor Tregs prior to allogeneic stem cell transplantation to determine whether the incidence of acute GVHD is reduced.

## Other Approaches

### α1-antitrypsin

α1-antitrypsin (A1AT), is a protease inhibitor produced by the liver and can inactivate serine proteases produced by myeloid cells and suppress their ability to produce pro-inflammatory cytokines. Pre-clinical studies have been performed which have revealed unique mechanisms for how A1AT influences acute GVHD. Marcondes et al., 2011 showed that A1AT could mediate protection by first demonstrating that it suppresses IL-32 and T cell proliferation *in vitro* (106). Utilization of an MHC-minor antigen model revealed that A1AT reduced several inflammatory cytokines including IL-1β and TNFα. This decrease in inflammatory cytokines resulted in a reduction in interstitial gastritis, crypt loss, and apoptosis in the duodenum, which ameliorated GVHD-induced mortality.

With respect to GVHD prevention, Gergoudis et al. performed a biomarker-guided preemptive study examining whether administration of A1AT could reduce the incidence of GVHD in patients deemed to be at high risk for steroid-resistant complications (107). Thirty patients that were identified as high risk for steroid refractory acute GVHD determined by a composite risk score that included measurement of Reg3α and ST2. Prior data have shown that these biomarkers in particular are predictive for the development of GI GVHD, making them surrogate candidates for prophylactic intervention. Results from this study were comparatively analyzed against a contemporaneous historical control population that did not receive A1AT therapy. Unfortunately, this study revealed that there was no reduction in GVHD incidence compared to the control group, indicating that A1AT administration had no impact on preventing the emergence of steroid refractory GVHD. Overall, while there has been some evidence that A1AT therapy could be beneficial for steroid resistant acute GVHD treatment (108), there have been no strict prophylaxis studies that have proven that A1AT can prevent GVHD arising in the GI tract.

### Histone Deacetylase Inhibition

Histone deacetylases (HDACs) play a key role in regulating gene transcription by acting as transcriptional repressors to remove acetyl groups and promote chromatin condensation (109). HDAC inhibitors are chemical compounds that irreversibly block the action of HDACs to uncoil condensed chromatin and allow for post-translational modifications of genes. In particular, HDAC inhibitors have been demonstrated to play a role as antitumor agents by inducing cell cycle arrest and apoptosis (110). Moreover, HDAC inhibitors have been utilized to treat various neurodegenerative diseases (111), to improve depressive behaviors and stabilize epileptic events (112). Recently, HDAC inhibitors have been highlighted for their ability to alleviate inflammation within the gastrointestinal tract (113) due to their ability to quell NF-ĸB-mediated cytokine release (114) and promote epithelial regeneration (115). Due to these properties, HDAC inhibition has been explored as a therapeutic strategy for ameliorating GI GVHD both preclinically and clinically (116–118).

Reddy and colleagues were the first to evaluate whether the HDAC inhibitor suberoylanilide hydroxamic acid (SAHA) could improve GVHD-induced morbidity and mortality in an MHC-mismatched murine model of the disease (119). They demonstrated that SAHA could reduce serum levels of proinflammatory cytokines TNFα, IL-1β, and IFN-γ. Moreover, SAHA limited severe villous blunting, crypt destruction and inflammation in the small intestine that was observed in vehicle treated mice, which resulted in improved survival. Importantly, these benefits did not seem to compromise GVL effects as they identified HDAC inhibitors as novel therapeutic agents for GVHD. A subsequent study performed by Reddy et al. expanded upon the mechanism of HDAC inhibition for GVHD by reporting that pretreatment of DCs with HDAC inhibitors could reduce TLR-mediated secretion of proinflammatory cytokines, increase indoleamine 2,3-dioxygenase (IDO) and suppress activation markers CD40 and CD80 (120). Furthermore, injection of DCs cells *ex vivo* with HDAC inhibitors before transplant was sufficient to protect mice from GVHD. These findings illustrate the prominent role HDACs play in regulating DC function to aggravate intestinal damage associated with GVHD. Another group corroborated the benefits of HDAC inhibition for GVHD by elaborating on its mechanism for protection of GVHD mice (56). Leng et al. identified that SAHA could limit GVHD-induced mortality by limiting TNFα and IL-1β levels through the phosphorylation of STAT1 in the liver and spleen. Whether or not HDAC inhibition-mediated prevention of STAT1 phosphorylation is an important mechanism for protecting the GI tract during GVHD was not evaluated.

The pre-clinical success observed with HDAC inhibition laid the foundation for testing whether this therapy could be beneficial for GVHD prophylaxis. Choi et al. performed a phase I/II trial to evaluate whether the HDAC inhibitor, vorinostat, could reduce the incidence of GVHD if administered 10 days before transplantation until day 100 in patients with high-risk hematological malignant disease who received stem cell grafts from matched related donors after reduced intensity conditioning (121). They found that vorinostat, in addition to standard GVHD prophylaxis was both safe and reduced the incidence of grades II-IV GVHD (22%) by day 100 compared to historical controls. However, of the patients that developed GVHD, most of them were reported to have GI GVHD. A second trial by the same group tested whether vorinostat, along with standard prophylactic agents, could prevent acute GVHD in recipients of unrelated stem cell grafts that received myeloablative conditioning (122). This study also identified that vorinostat was safe and resulted in grade II-IV GVHD occurring in 22% of patients, with only 8% exhibiting grade III-IV GVHD. Moreover, they performed correlative analyses in PBMCs from these patients to find that IL-6, Reg3α, and ST2 (all markers associated with GI GVHD) correlated with reduced GVHD in patients at day 30 after transplant. This study also revealed that only 11% of patients displayed GI GVHD at day 100. Together, these trials provide evidence that vorinostat has promise for the prevention of GVHD in the GI tract.

A second HDAC inhibitor, panobinostat, was recently evaluated both in a phase I trial for GVHD treatment (123) and in a phase II trial for GVHD prophylaxis (124). In the prophylactic trial, intervention with panobinostat began at -5 days before transplant and was continually administered for 28 weeks in patients with acute myeloid leukemia (n= 18), myelodysplastic syndrome (n = 13) and other malignancies (n = 8). The cumulative incidence rate of grade II-IV acute GVHD at 100 days was only 18.4% and the one-year overall survival was 89.5%. Importantly, of the patients who developed acute GVHD and received the full treatment of panobinostat, none developed greater than grade I GI GVHD. In addition, they observed a decrease in plasma IL-6 levels in treated patients at day 90 compared to controls but did not witness any differences in Reg3α and ST2 at day 28, unlike the vorinostat study. Overall, reports from clinical trials utilizing vorinostat and panobinostat indicate that HDAC inhibition could be an appropriate preventative strategy for GI tract GVHD.

### Proteasome Inhibition

Proteasomes are large catalytic protein complexes that cleave and degrade misfolded, damaged or erroneous proteins into peptides (125). They can also play a role in inducing activation of NFĸB-dependent signaling pathways that are responsible for preventing apoptosis and promoting the release of proinflammatory cytokines. Due to these capabilities, unregulated proteasome activity has been demonstrated to correlate with the severity of autoimmune diseases and cancer (126). Proteome inhibitors have been demonstrated to be effective as anti-tumor agents (127) and to reduce NFĸB –mediated inflammation in models of psoriasis (128) and asthma (129) as well. For these reasons, there has been interest in investigating whether proteasome inhibition could be efficacious for GVHD prophylaxis.

Bortezomib, a boronic acid dipeptide derivative, was the first proteasome inhibitor to be approved by the US Food and Drug Administration (FDA) in 2003 (130) and was initially clinically approved as a therapy for multiple myeloma patients due to its growth-inhibitory and anti-apoptotic effects (131). Sun and colleagues performed seminal experiments to test whether bortezomib could have prophylactic effects for acute GVHD (132). They found that bortezomib could promote the apoptosis of alloreactive T cells *in vitro* and protect mice from acute GVHD *in vivo* without adversely affecting donor reconstitution when administered at the time of transplant. Subsequently, Vodanovic-Janovic et al. evaluated whether administration of bortezomib could protect mice from GVHD (133). They reported that early post-transplant therapy with bortezomib improved GVHD-free survival without compromising donor engraftment; however, extended administration of bortezomib exacerbated pathological damage in the colon and resulted in early mortality due to gut toxicity. This study indicated that while early post-transplant proteasome inhibition may be beneficial, more protracted administration exacerbated GVHD-induced immune-mediated damage in the GI tract.

Sun et al. corroborated these findings and demonstrated that prolonged bortezomib administration increased serum levels of TNFα and IFNγ in multiple murine models of GVHD and led to early mortality (134). The detrimental effect of bortezomib on allogenic T cells appeared to be CD4 mediated and TNFα dependent as mice transplanted with TNFα deficient donor CD4 T cells were resistant to the toxic effects of bortezomib. More recently, Li and colleagues (135) reported that early doses of bortezomib on days 0 and 1 after transplant prevented pathological damage in the GI tract and improved survival. This benefit corresponded with decreased serum levels of IL-2, TNFα and IFNγ. Overall, these preclinical studies indicated that bortezomib administration could protect the GI tract from GVHD but that this was schedule dependent.

Based on preclinical results indicating a protective role of bortezomib administration for acute GVHD, Koreth and colleagues conducted a phase I/II trial to test whether a short course of bortezomib could be an applicable intervention for GVHD prophylaxis following transplantation from HLA-mismatched unrelated donors for patients with hematologic malignancies (136). Of the patients who were given bortezomib, 22% displayed grade II to IV acute GVHD at day 180 and importantly, these patients did not experience augmented GI toxicity. Two subsequent phase II trials were performed by Koreth and colleagues to evaluate the prophylactic effects of bortezomib on GVHD. The first involved administration of short-course bortezomib for patients who underwent myeloablative conditioning and included both HLA-matched and HLA-mismatched donors (137). Bortezomib was effective in generating a low incidence of grade II to IV acute GVHD involving the skin, liver and/or lower GI tract with only 12% of patients displaying grade III to IV acute GVHD. The second was an open-label three-arm phase II randomized control trial in patients who received reduced intensity conditioning and allogenic transplants lacking HLA-matched donors (138). Unfortunately, this trial reported that bortezomib-based regimens did not lower GVHD incidence compared to control regimens. A more recent phase II randomized trial compared multiple interventions, one including bortezomib in addition to standard immunosuppression, to evaluate its role in GVHD prophylaxis, which involved both HLA-matched and HLA-mismatched donors (79). Similarly, this study demonstrated that bortezomib had no beneficial effect on reducing the incidence of grades II-IV acute GVHD when compared to standard immune suppression alone. While none of these studies specifically examined the GI tract for organ-specific protective effects, the lack of any overall reduction in acute GVHD argues against any protective effect in this tissue site.

## Conclusions

The GI tract is the major site of morbidity and mortality associated with the development of acute GVHD. Unfortunately, a significant percentage of patients fail to respond to first line therapy with corticosteroids and require second line therapy for steroid refractory disease. Outcomes for these patients is significantly worse and a substantial proportion of these patients ultimately do not respond to salvage therapy. In addition, patients who develop GI GVHD often require hospitalization and are at risk for secondary infections due to compromised epithelial barrier integrity, which adversely impacts quality of life and can also result in premature fatality. Consequently, prevention of this complication, particularly within the GI tract, is critically vital to improve overall treatment outcomes and should be a primary goal of GVHD prophylaxis strategies.

To that end, preclinical studies have identified that inhibition of inflammatory cytokine pathways, blockade of gut homing molecules that are expressed on the surface of alloreactive donor T cells, and reconstitution of regulatory pathways as potential therapeutic strategies that have shown promise and led to translation in human clinical trials. Unfortunately, many of these strategies, while promising in animal studies, have not translated well into the clinic. Reasons for this are not entirely clear but are likely multifactorial and related to limitations of mouse models that do not fully replicate the complexity of human allogeneic stem cell transplantation with respect to recipient age, stem cell source, conditioning regimen intensity, and MHC disparity which all impact GVHD severity. In other cases, some of these approaches have only recently entered clinical trials for GVHD prophylaxis (e.g., blockade of α4β7 integrin and IL-23 signaling) so the verdict is still out on whether they will be efficacious for prevention of GI tract GVHD. To date, blockade of IL-6 signaling, administration of Treg infusions, and histone deacetylase inhibition have reported clinical outcomes in which there appears to be a reduction in GI tract GVHD; however, definitive data are still lacking with these approaches. Thus, additional investigations are required to clearly identify effective prophylactic strategies that will ameliorate toxicity to this important tissue site, and secondarily lead to an improvement in overall transplant outcomes.

## Author Contributions

AR and WD wrote and edited the manuscript. All authors contributed to the article and approved the submitted version.

## Funding

This research was supported by grants from the National Institutes of Health (HL064605, HL126166, and HL139008).

## Conflict of Interest

The authors declare that the research was conducted in the absence of any commercial or financial relationships that could be construed as a potential conflict of interest.

## Publisher’s Note

All claims expressed in this article are solely those of the authors and do not necessarily represent those of their affiliated organizations, or those of the publisher, the editors and the reviewers. Any product that may be evaluated in this article, or claim that may be made by its manufacturer, is not guaranteed or endorsed by the publisher.
